# Co-existence of Thrombotic Thrombocytopenic Purpura and Megaloblastic Anaemia: A Case-Based Review

**DOI:** 10.31138/mjr.33.2.241

**Published:** 2022-06-30

**Authors:** Partisha Gupta, Sakir Ahmed, Nikunj Kishore Rout, Chaitanya Yelisetti, Ranjita Panigrahi, Pradip Kumar Behera, Krishna Padarabinda Tripathy, Sudhansu Sekhar Panda

**Affiliations:** 1 Department of Internal Medicine, Kalinga Institute of Medical Sciences (KIMS), KIIT University, Bhubaneswar, India,; 2 Department of Clinical Immunology & Rheumatology, Kalinga Institute of Medical Sciences (KIMS), KIIT University, Bhubaneswar, India,; 3 Department of Nephrology, Kalinga Institute of Medical Sciences (KIMS), KIIT University, Bhubaneswar, India,; 4 Department of Pathology, Kalinga Institute of Medical Sciences (KIMS), KIIT University, Bhubaneswar, India

**Keywords:** TTP, microangiopathic haemolytic anaemia, megaloblastic anaemia, thrombocytopenia

## Abstract

**Introduction:**

Thrombotic thrombocytopenic purpura is a rare and fatal thrombotic microangiopathy characterised by a pentad of microangiopathic haemolytic anaemia, thrombocytopenia, renal abnormalities, neurological abnormalities, and fever. Due to ineffective erythropoiesis, vitamin-B12 deficiency may rarely present as haemolytic anaemia.

**Case report:**

We report a case of a 42-year-old vegetarian female presenting as vitamin B12 deficiency anaemia found to have concomitant TTP, responding to plasmapheresis, corticosteroids, and rituximab therapy.

**Discussion:**

In this case of vitamin B12 deficiency with co-existent TTP, we hypothesise vitamin B12 deficiency as a contributory or precipitating factor for TTP. We reviewed similar cases in the literature to support this hypothesis. Timely detection of TTP and the initiation of treatment is of utmost importance as TTP has a high mortality when left untreated. The possible relationship with Vitamin B12 deficiency needs further exploration.

## INTRODUCTION

Thrombotic thrombocytopenic purpura (TTP) is a rare and potentially fatal thrombotic microangiopathy. It is specifically related to a severe deficiency in ADAMTS13 (a disintegrin and metalloprotease with thrombospondin type 1 repeats, member 13), the specific von Willebrand factor-cleaving protease. TTP is characterised by pentad of microangiopathic haemolytic anaemia (MAHA), thrombocytopenia, renal abnormalities, neurological abnormalities, and fever.^1^ The classical pentad is an advanced feature, and for diagnosing TTPa clinician should never wait for the full pentad to manifest. Diagnosis can be made in the presence of microangiopathic haemolytic anaemia and one other feature with elimination of other causes.

TTP can be classified as genetic and acquired. ADAMTS13 deficiency is most frequently acquired via ADAMTS13 autoantibodies, but rarely, it is inherited via mutations of the ADAMTS13 gene.^2^ TTP could be secondary to infections (HIV-1, etc.), pregnancy, bone marrow transplantation, and medication use (anti-viral such as acyclovir, quinine, platelet aggregation inhibitors such as ticlopidine, clopidogrel, prasugrel). Many a times, past infection may not be evident in all such cases.

Here we report a case of a 42-year-old female with vitaminB12 deficiency ultimately found out to have concomitant TTP. We hypothesise that vitamin B12 deficiency might have been a contributory or even a precipitating cause of TTP in this case. Since the features of both can be overlapping, an association may have been overlooked previously.

## SEARCH STRATEGY

In view of our hypothesis, we searched PubMed/MEDLINE and Scopus for “thrombotic thrombocytopenic purpura” in various combinations with “vitamin B12 deficiency” or “megaloblastic anaemia” for relevant articles as per standard recommendations for biomedical reviews^3^.

## CASE PRESENTATION

A 42-year-old female, teacher by profession, presented with chief complaints of high-grade fever and easy fatigability for eight days. No history of cough, headache, abdominal pain, decreased urination, active bleeding, or any recent drug intake. She had no co-morbidities; regular menstrual cycle, and consumed a vegetarian diet. At the time of presentation, she was conscious and oriented to time, place, and person. Vitals were stable. Pallor was present, and petechiae was visible in both the lower limbs. Lymph nodes were non-palpable. Per abdominal examination revealed no hepatosplenomegaly. Nervous system, cardiovascular, and respiratory system examination did not reveal any significant abnormality.

**Figure 1.**
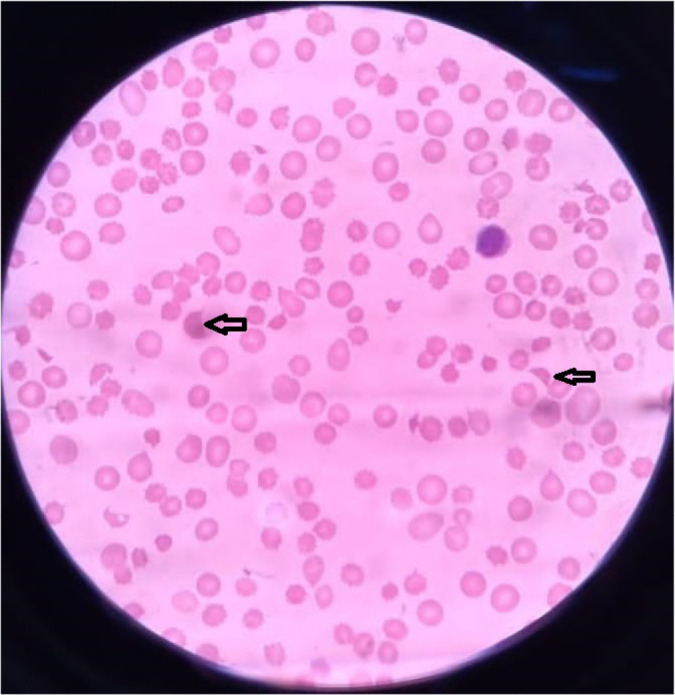
Peripheral smear showing polychromatic cells with schistocytes (black arrows) and nucleated red blood cells.

During the hospital stay, initial laboratory reports revealed bicytopenia with the total leucocyte count 9.9 × 10^3/^μL, haemoglobin 4.4 g/dL, mean corpuscular volume of 96.6 fL/um^3^, and platelets of 8 × 10^3^/μL. The peripheral smear showed reduced red cell mass, moderate anisocytosis, anisochromia, presence of macrocytes, normocytes, and polychromatic cells with schistocytes and nucleated red blood cells as shown in **Figure 1** below. Renal function test and serum electrolytes were within normal limits. The reticulocyte proliferation index was 2.7. Her liver function test revealed total bilirubin of 3.74 mg/dL, indirect bilirubin of 2.06 mg/dL, aspartate transaminase was 168 U/L and alanine transaminase 88 U/L suggestive of haemolysis. Prothrombin time (11.1 sec) with international normalized ratio of 1.3, and partial thromboplastin time (21.6 sec) and were within normal limits. Direct Coombs’ test was negative. Since the patient was strict vegetarian, vitamin B12 levels were quantified to be 144 pg/mL (reference range 211–911pg/mL). Serum lactate dehydrogenase was 1442.34 U/L (Reference range <250U/L). Bone marrow aspiration revealed hypercellular marrow with megaloblastoid picture. The patient was promptly started on intravenous methyl-cobalamin therapy and oral folic acid.

However, on the fourth day of hospital stay, the patient developed three episodes of generalised tonic-clonic seizures and became disoriented. Non-contrast computed tomography of the brain ruled out intracranial bleeding. There was rising LDH, without improvement in the platelet count or the haemoglobin level. In view of the neurological involvement along with thrombocytopenia, fever, and microangiopathic haemolytic picture of the blood smear, and lack of response to vitamin B12 therapy, a possibility of TTP was considered. PLASMIC score^7^ was 8 suggesting a high probability of TTP. Anti-nuclear antibody was negative, and complement levels were normal.

On the fourth day, plasmapheresis was initiated. Simultaneously, pulse methylprednisolone was also initiated, and after 3 days the patient was shifted to high dose oral steroids (1mg/kg). The patient received a total of 5 cycles of plasmapheresis. The general condition of the patient improved but continued to fluctuate, and the platelet count and haemoglobin level increased to 78 × 10^3^ u/L and 8g/dL, respectively. ADAMTS-13 activity later came out to be 7% (Reference range- >10%), and anti-intrinsic factor antibody was negative. Patient was given 2 doses of Rituximab 14 days apart to prevent relapse. Patient responded well to therapy and clinical and haematological parameters improved within 7 days. Neurological features improved by day 10 and there were no more seizures. Doses of steroids was tapered gradually after completion of 6 weeks.

## DISCUSSION

Initially the diagnosis of vitamin B12 deficiency had seemed viable as she was a young vegetarian female, with bicytopenia, haemolysis, and a megaloblastic bone marrow picture. This was supported by the serum vitamin B12 levels. Presence of schistocytes have been reported previously in Vitamin B12 deficiency anaemia. However, the absence of response to parental B12 therapy and the neurological involvement pointed towards the diagnosis of TTP. This was supported by the PLASMIC score, the response to immunosuppression and the reduced ADAMTS-13 activity of 7%.

In the review of literature, the cases reported as vitamin B12 deficiency mimicking TTP-like picture^8–11,13–17^ are summarized in **Table 1**. The majority of patients had presented with microangiopathic haemolytic anaemia (MAHA). Most have been labelled as pseudo-thrombotic microangiopathy. The mechanism of vitamin B12 deficiency linked with MAHA is thought to be due to acute hyperhomocysteinemia. ^4^ Homocysteine down-regulates the activity of glutathione peroxidase, leading to the intracellular accumulation of reactive oxygen species, and destruction of red cells. Therefore, the homocysteine induced oxidative stress can lead to microangiopathy,^5^ which can deviate the pathological picture towards haemolytic anaemia in the absence of other diagnostic clues. The ‘pseudo’ term seems appropriate because in the case-based review at least 4 of them improved on vitamin B12 supplementation only, others were also administered low dose immunosuppressives.

**Table 1.** Case reports of presentation of vitamin B12 deficiency similar to or co-existent with TTP.

**Ref No**	**Age/Sex**	**Background illness**	**Clinical features**	**Final diagnosis**	**Treatment**	**Outcome**
8	42/F	Nil	Presented with fatigue and weakness. With profound anaemia and thrombocytopenia	Pernicious anaemia secondary to vitamin B12 deficiency	Plasma exchange given once. Vitamin B12 supplementation	Responded well to vitamin B12 supplementation.
9	74/M	Nil	Exertional dyspnoea, fatigue and anorexia with pancytopenia, unconjugated jaundice	Pseudo-TMA with Pernicious anaemia due to vitamin B12 deficiency	Plasma therapy, Parenteral B12 supplementation	Not available
10	42/M	Previously treated hepatitis C virus infection	Worsening dizziness and near falls, malaise, blurry vision, and ataxia. Marked anisopoikilocytosis, presence of tear drop cells, schistocytes, and hyper segmented neutrophils.	Pseudo-TMA with pernicious anaemia due to vitamin B12 deficiency	Plasmapheresis thrice, vitamin B12 supplementation	Responded well to vitamin 12 therapy with complete resolution of symptoms and laboratory parameters
11	14/M	Nil	One-week tiredness with pallor, hyperpigmented palms, splenomegaly	Vitamin B12 deficiency with pseudo-TMA	Vitamin B12 supplementation	Good response to the treatment
12	46/M	Nil	History of haemoptysis and haematuria for one day	Acquired TTP in the setting of pernicious anaemia	Plasmapheresis	Resolution of symptoms and biochemical parameters
13	77/M	Nil	Altered sensorium, renal insufficiency, and thrombocytopenia. Anisopoikilocytosis with schistocytes.	Pseudo-TMA with vitamin B12 deficiency anaemia	Plasmaphereses, parenteral B12 supplementation for life	Complete resolution of symptoms and laboratory parameters 3 weeks post-discharge
14	65/M	History of hepatitis- C, Alcoholic	One-month history of increasing fatigue, with diffuse ecchymosis of both forearms. 4% schistocytes, 2+ tear drop cells, and hyper-segmented neutrophils in smear.	Pseudo-TMA with vitamin B12 deficiency	Parenteral B12 supplementation	Complete resolution of anaemia 4 months later
15	46/m	Chronic alcoholic, chronic smoker	Two-month history of progressive fatigue, arthromyalgia, upper finger paraesthesia, recurring headache, and left ear tinnitus. Schistocytes and anisopoikilocytosis in smear.	Pseudo-TTP secondary to vitamin B12 deficiency	Parenteral B12 supplementation	Not available
16	42/M	Diabetic ketoacidosis	Presented with complaints of presyncope and diabetic ketoacidosis with anaemia, thrombocytopenia, and schistocytes.	Pseudo-TTP secondary to vitamin B12 deficiency	Plasmapheresis and steroids were discontinued on day 5 due to non-response to therapy; Parenteral B12 supplementation given	Improved marrow function after 3–4 weeks
17	52/M	Nil	14 days of dyspnoea, general weakness, weight loss, and a sore tongue. Schistocytes +.	Pseudo-TTP secondary to vitamin B12 deficiency anaemia	2 units of packed red cells, parenteral B12 supplementation for life	Resolution of biochemical parameters within 9 days of therapy

In our case, as well as at least one other case,^12^ there is evidence of co-existence of TTP and vitamin B12 deficiency. Thus, we hypothesise that vitamin B12 deficiency could be a contributory or even a precipitating cause of TTP. Since the features of both can be overlapping, an association may have been overlooked previously. Vitamin B12 has antioxidant properties, and the deficiency of vitamin B12 is associated with elevated inflammatory markers, which includes interleukin-6 (IL-6), malondialdehyde, and high sensitivity C-reactive protein.^6^ It is interesting that IL-6 in-vitro models revealed that it can be a direct catalytic inhibitor of ADAMTS13.^7^ Therefore, rarely the oxidative stress-induced IL-6 production in severe vitamin B12 deficiency can reduce ADAMTS13 activity, leading to the accumulation of unusually large multimers of von Willebrand factor causing platelet aggregation and thrombi.

TTP and pseudo-thrombotic microangiopathy (pseudo-TMA) has similar clinic-pathological picture. However, in contrast to TTP, the reticulocyte count is low in pseudo-TMA due to the unavailability of vitamin B12 for the compensatory increase in erythropoiesis. ADAMTS-13 activity is normal in pseudo-TMA, while in hereditary or acquired TTP it should be <10%. Pseudo-TTP responds well only to vitamin B12 supplementation without any need of plasma exchange, in contrast to TTP where plasmapheresis is the mainstay of treatment. Without treatment, TTP has a mortality rate approaching 90%. With the timely institution of therapeutic plasma exchange mortality decreases to about less than 10%.^18^

While diagnosing possibility of TTP, the PLASMIC score developed by Bendapudi et al.^19^ is a useful predictive tool for assessing the likelihood of severe ADAMTS13 deficiency in patients with TMA.

Early TTP-like features in vitamin B12 deficiency maybe reversed by supplementation, but once full TTP develops, we are of the opinion that both immunosuppression and vitamin B12 supplementation may be required in such overlap situations.

We acknowledge the limitation that the current evidence in support of our hypothesis is limited. However, we feel this may be an important area to be explored.

## CONCLUSION

TTP is a life-threatening condition that has a high mortality if left untreated. Vitamin B12 deficiency, commonly due to pernicious anaemia is known to cause pseudo-thrombotic microangiopathy responding well to vitamin B12 supplementation. However, there is a possibility that vitamin B12 deficiency could be a contributory or a precipitating factor for TTP, and its co-existence could have an overlapping presentation proving to be a dilemma for the clinician.

## ABBREVIATIONS

ADAMTS13: a disintegrin and metalloprotease with thrombospondin type 1 repeats, member 13
HIV: Human immunodeficiency virus
MAHA: Microangiopathic haemolytic anaemia
TTP: Thrombotic thrombocytopenic purpura
